# Microbiota and Human Reproduction: The Case of Male Infertility

**DOI:** 10.3390/ht9020010

**Published:** 2020-04-13

**Authors:** Rossella Tomaiuolo, Iolanda Veneruso, Federica Cariati, Valeria D’Argenio

**Affiliations:** 1 KronosDNA srl, spinoff of Università Federico II, 80133 Napoli, Italy; rossella.tomaiuolo@unina.it (R.T.); cariati@ceinge.unina.it (F.C.); 2 Department of Molecular Medicine and Medical Biotechnologies, Federico II University, Via Sergio Pansini 5, 80131 Napoli, Italy; io.veneruso@studenti.unina.it; 3 CEINGE-Biotecnologie Avanzate scarl, Via Gaetano Salvatore 486, 80145 Napoli, Italy; 4 Department of Human Sciences and Quality of Life Promotion, San Raffaele Open University, 00166 Roma, Italy

**Keywords:** microbiota, human microbiome, human reproduction, male infertility, semen microbiome

## Abstract

The increasing interest in metagenomics is enhancing our knowledge regarding the composition and role of the microbiota in human physiology and pathology. Indeed, microbes have been reported to play a role in several diseases, including infertility. In particular, the male seminal microbiota has been suggested as an important factor able to influence couple’s health and pregnancy outcomes, as well as offspring health. Nevertheless, few studies have been carried out to date to deeper investigate semen microbiome origins and functions, and its correlations with the partner’s reproductive tract microbiome. Here, we report the state of the art regarding the male reproductive system microbiome and its alterations in infertility.

## 1. Introduction

Almost all human tissues host microbes that have been widely recognized as important players for human health and disease status [1,2]. Indeed, microbes have also been found in several body niches previously considered to be sterile, and may influence humans’ physiology and/or pathology already before birth [3]. As a consequence, the study of microbial genomes, i.e., the microbiome, has gathered increasing interest, with the aim to better understand and define the microbial contribution to human diseases, thus opening novel opportunities for diagnosis and therapy [1,2,3].

Infertility has become an important health issue worldwide [4]. Indeed, changes in lifestyle and the increased age of both partners at first conception have contributed to the dramatic increase of infertility incidence worldwide [4]. Even though several causes of infertility have been described so far, and it seems to be established that the reproductive systems of both partners have to work in a coordinated manner, the molecular mechanisms underlying infertility have not yet been clearly elucidated.

Microbiota have been suggested to also play a role in this context. In particular, while several studies have been carried out focusing on the female reproductive system [5,6,7], less is known about male microbiota and its influence on reproduction and fertility.

Recent studies have shown that the seminal microbiota may not only play a role in the maintenance of men’s reproductive health, but may also be related to the health of the couples and of their offspring through the transfer of microorganisms [8,9,10,11,12]. In particular, alterations of the seminal microbiome have been associated with some sperm features, i.e., altered motility and increased DNA fragmentation [13,14], fueling the interest for deeper investigations in this emerging field of metagenomic studies.

Taking into account all the above, this review aims to summarize the current state of the art regarding semen microbiota composition and its role on men’s health. In particular, evidence suggesting a link between semen microbiome dysbiosis and male infertility will be also reviewed.

## 2. Materials and Methods

PubMed was examined for indexed articles in English published between 2010 and 2020 using the following as keywords: “male infertility and microbiota”, “male infertility and metagenomics”, “male reproductive system microbiome”, and “semen microbiome”. These temporal limits were chosen to focus on the more recent and updated papers on these topics, also taking into account that metagenomic boom raised in the last ten years and the interest in semen microbiome evaluation has begun more recently. Moreover, a manual search for the oldest references mentioned in the found articles was also carried out.

## 3. The Combined Multiple Composition of Semen Microbiome

Male semen, being a mixture of sperm and secretions of sexual accessory glands, containing nutrients (such us lipids, proteins, glycans and inorganic ions), is an ideal environment for microbe growth [15,16,17]. While in the past it was thought that the presence of bacteria in the semen was a sign of infection, the use of next generation sequencing-based approaches has revealed that the human semen is not sterile and hosts a specific microbiota [8,9]. To date, the functions of this resident microbiota in maintaining a healthy status in men have not been completely understood, and it seems that the semen microbiota could be involved in immune system reactions [12]. In addition, currently the origin of this microbiota is still unclear. Kermes et al., comparing the bacterial communities of semen and urethra, showed a low similarity between these two body niches, suggesting that the seminal microbiota may originate from the upper genital tract [18]. The most recognized hypothesis is that the seminal microbiome may have a combined multiple origin from different urogenital tissues and from the gut, mouth, blood and vagina [10,18,19,20].

Several studies have reported a high inter-individual variability identifying different community-types with different dominant bacteria [8,21]. In particular, Hou et al., based on microbial communities clustering, were able to discriminate six different groups among healthy semen donors [21]. Chen et al., studying azoospermic patients, found a prevalence of the *Lactobacillus* genus [22], while another study by Monteiro et al., showed a low abundance of *Lactobacilli* [9]. Mandar et al., comparing the semen microbiome of patients with prostatitis versus controls, found a higher amount of *Lactobacilli*, and in particular *L. iners*, in the healthy subjects group [23]. *Lactobacilli* have been identified in the human vagina, oral cavity and gastrointestinal tract, where they exert protective functions. Thus, it is conceivable that they may also have a similar role in the male genital tract [12]. According to this hypothesis, *Lactobacillus*-predominant semen has been reported to have higher quality with respect to *Prevotella*-or *Pseudomonas*-predominant semen [19]—with *Lactobacilli* being able to prevent sperm lipid peroxidation, and to preserve sperm motility and viability [24].

## 4. The Role of Semen Microbiome on Reproduction

Infertility affects one in seven couples worldwide and its diagnosis and treatment still require a long time and high costs, and may be frustrating for couples [4]. Several pre-testicular, testicular and post-testicular causes of male infertility have been reported to date [4]. These include genetic, immunological, infective, and anatomical factors that are all able to promote inflammatory reactions in the male genital tract [4,23,25]. The inflammatory status, in turn, has been linked to a poor semen quality and, finally, to male infertility [9,26,27].

In particular, it has been established that microorganisms can impair spermatozoa functions through several mechanisms. Interestingly, it has been suggested that this toxic effect may not only be mediated by inflammatory cytokines or by enhancing the production of oxygen reactive species [28,29,30], but microbes seem to also be able to directly interact with the spermatozoa by adhesion to these cells, or by releasing soluble factors which are able to affect sperm motility or promote apoptosis [9,26,27]. However, the molecular mechanisms through which urogenital infections or specific bacteria are able to impair host physiology, inducing semen quality alterations and, consequently, typical infertile features, are still poorly understood [9].

**Figure 1:**
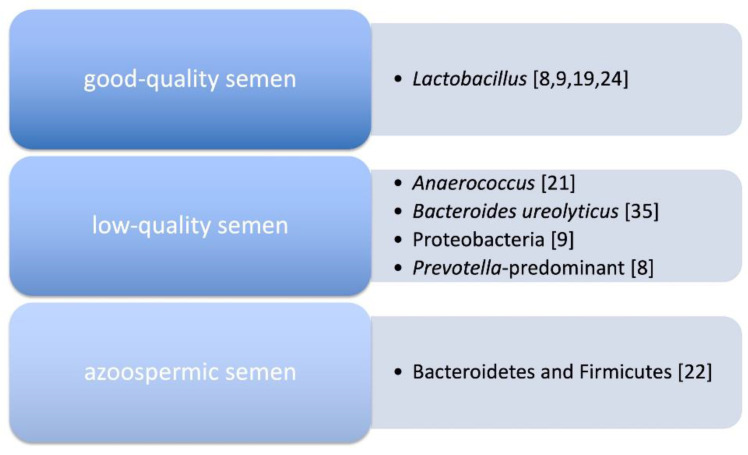
Correlation between semen quality and its microbiota. References are reported in parentheses.

For many years, it has been reported that routine plate culture assessment in infertile men gives positive results for aerobic bacteria growth in 15–100% of the analyzed cases, but similar results have been found also in healthy and fertile men [14,31,32,33]. Thus, anaerobic bacteria have been also investigated. In this context, Rehewy et al. cultured the semen obtained from both infertile and fertile men and found more viable bacteria in the infertile subjects [34]. Balmelli et al., by analyzing 3196 infertile men, identified a correlation between the presence of the *Bacteroides ureolyticus* and the increased presence of short-tailed spermatozoa [35]. More recently, next generation sequencing-based studies have been carried out to highlight microbial features in semen samples specifically related to infertility [8,9,21,22]. Hou et al. did not find significant differences among controls and infertile men, but were able to identify a correlation between the presence of the *Anaerococcus* and a reduced sperm quality, suggesting that this bacterium may play a role in infertility [21]. Monteiro et al., analyzing 118 semen samples with different phenotypic features, found that the seminal microbiota in presence of hyperviscosity and oligoasthenoteratozoospermia had an increased abundance of Proteobacteria and a reduction in Lactobacillus with respect to the controls, thus suggesting that these microbial alterations may be related to a poor reproductive outcome [9]. However, Chen et al. found a general reduction in biodiversity and an increase in Bacteroidetes and Firmicutes in azoospermic patients with respect to healthy men [22]. Weng et al. analyzed 96 infertile men and found three different clusters: *Lactobacillus*-predominant, *Pseudomonas*-predominant and *Prevotella*-predominant, the latter being associated with low-quality sperm [8]. However, these studies were not able to put forward conclusive remarks regarding the role of semen microbiota in infertility, and additional, well-designed studies are required to better address this issue. Nonetheless, taken together, they suggest an intriguing association between microbes and some sperm parameters related to male infertility (Figure 1).

If confirmed by future studies, this association may provide novel insights in the pathogenesis of infertility and open the way to novel therapeutic approaches to ameliorate the outcome of infertile couples.

It has been well established that a successful infertility treatment cannot be achieved if just one of the partners is investigated, since infertility has to be considered as the final result of a combinatory process involving both male and female factors [4]. This concept applies also to the genital tract microbiome. Indeed, it has been shown that unprotected sexual intercourse can determine an exchange of microbes [21]. In particular, bacteria can not only be shared among partners, but it seems that each member of a couple can influence the microbiome composition of the other, and that several factors, i.e., age at sexual debut, contraceptive use, frequency and mode of sexual activity are important modifying factors [19,21,23,36].

In this context, several studies have been carried out to verify the effects of the male genital microbiota on that of the female [37,38,39,40,41,42,43,44,45]. In particular, it has been assessed that frequent sexual intercourse, multiple sexual partners, and uncircumcised male partners can be related to vaginal microbiota alteration and also to bacterial vaginosis [37,38,39,40,41,42]. Vodstrcil et al. evaluated the effects of sexual activity on the composition of the vaginal microbiota and found a positive correlation with the presence of *Gardnerella vaginalis*, thus suggesting the sexual transmission of microbes [43]. However, other studies did not highlight a significant correlation between the vaginal microbiota and sexual intercourse [44,45].

In the same way, other studies aimed to evaluate the effect of the female genital microbiota on that of the male [8,21,23,36]. Mandar et al. (2018) showed that men without sexual experience had lower bacterial load and diversity in their semen compared to sexually experienced men, thus supporting the hypothesis that female genital microbiota can influence that of the male [36]. Indeed, vaginal bacterial taxa have been described in semen [8,21]. In particular, Mandar et al. (2017) highlighted a correlation between male age and the kind of vaginal bacteria in semen, probably due to the different, age-dependent sexual activity frequency [23].

Another point which is still poorly investigated and worthy of exploration is represented by endometrial microbiota modifications during the menstrual cycle and their potential influence on the partner’s semen microbiome. Since it is known that hormones influence the microbiota [46,47], it will be expected that estrogen fluctuations during the menstrual cycle may impact the uterine microbiota composition. According to this hypothesis, Chen et al. analyzing 95 women in the proliferative and secretory phases, found that the *Propionibacterium acnes* was more abundant in the uterus during the secretory phase [48]. Considering that it has been assessed that the female reproductive system microbiota can influence the semen microbiome of their partners [8,21,23,36], it is appropriate to hypothesize that women’s menstrual cycle may also play a role. Further studies are required to address this issue.

To date, few studies have been carried out to assess a correlation between male and female microbiomes [19,49]. Wittemer et al. analyzed 951 couples undergoing in vitro fertilization procedures and, by culturing endocervical, vaginal and seminal microbiotas, found not only a lower implantation rate in women with endocervical bacterial growth, but also a reduced pregnancy rate and an increased number of miscarriages in the presence of positive vaginal and/or seminal cultures [49]. Mandar et al. analyzed pre- and post-coital vaginal samples and semen samples from the partner, and found that semen communities were significantly different and had a lower bacterial richness compared to vaginal samples [19].

Interestingly, *Chlamydia trachomatis* is the most common sexually transmitted infection worldwide and has also been associated with infertility, even if the underlying mechanisms are still not completely understood [50]. In particular, an association between microbiota dysbiosis and the risk of *Chlamydia* infections has been reported [51]. Wiesenfeld et al. found that women with bacterial vaginosis, who had recent exposure to a male partner with chlamydial urethritis, have a higher risk of *Chlamydia* infection [52]. It has been proposed that bacterial vaginosis, through the reduced presence of Lactobacillus spp. and the increased abundance of indole-producing microbes, may create an environment facilitating chlamydial infection [53,54]. This may be another mechanism of microbial spread between male and female partners’ microbiotas, as well as another link between couples’ reproductive system’s microbiota and infertility.

In addition to these microbial exchanges between partners, it has been hypothesized that the paternal microbiome may also affect the offspring [12,55,56,57,58]. While it has been established that the intrauterine environment can influence the future adult health, the mechanisms through which the father can exert this effect are still unknown. It has been supposed that the fathers may influence their offspring through the seminal microbiome [55]. How this semen microbiome is able to influence and modify the offspring is still under investigation. Pan et al. suggest that the semen microbiome may affect offspring methylome and transcriptome, inducing persistent phenotypic features [56]. The possible effects on immune system modulation have also been hypothesized: Sisti et al. showed that the longer the exposure of the female to the semen of their partner (as occurs in long-term relationships), the higher the rate of regulatory T cells development, thus limiting maternal anti-fetal immune reactions [57]. Moreover, Lannon et al. assessed that the semen microbiome is able to influence the vaginal microbiome and, in turn, influence the ability of semen to overcome the cervical barrier and colonize the uterus [58].

## 5. Diet Supplementation and Semen Microbiota

Considering all the increasing evidence regarding the role of the seminal microbiome in male health and in several diseases, including infertility, it is not surprising that treatments able to target the microbiome are becoming an intriguing option. Plummer et al. have demonstrated that antimicrobial drugs in the male partners of women with bacterial vaginosis were able to exert beneficial effects reducing disease recurrence [42]. In addition, prebiotics supplementation has shown the ability to ameliorate some semen parameters in both animal models and humans [59,60,61,62]. Dardmeh et al. found that *Lactobacillus rhamnosus* PB01 (DSM 14870) supplementation was able to improve sperm kinematic parameters in mice [59]. Inatomi et al. showed that *Bacillus amyloliquefaciens* TOA5001 ameliorated the semen quality of male broiler breeders [60]. In humans, Valcarce et al. assessed that a 6-week supplementation with *Lactobacillus* and *Bifidobacterium* improved sperm motility and reduced the percentage of sperm DNA fragmentation in asthenozoospermic males [61]. Maretti et al. verified that the daily administration of *Lactobacillus paracasei*, arabinogalactan, fructo-oligosaccharides, and l-glutamine over a period of 6 months ameliorated sperm count and motility, and reduced the rate of atypical forms, in comparison to the placebo-receiving group [62]. These evidences, if confirmed by larger studies, may pave the way for novel therapeutic strategies for fertility issues.

## 6. Conclusions

**Figure 2:**
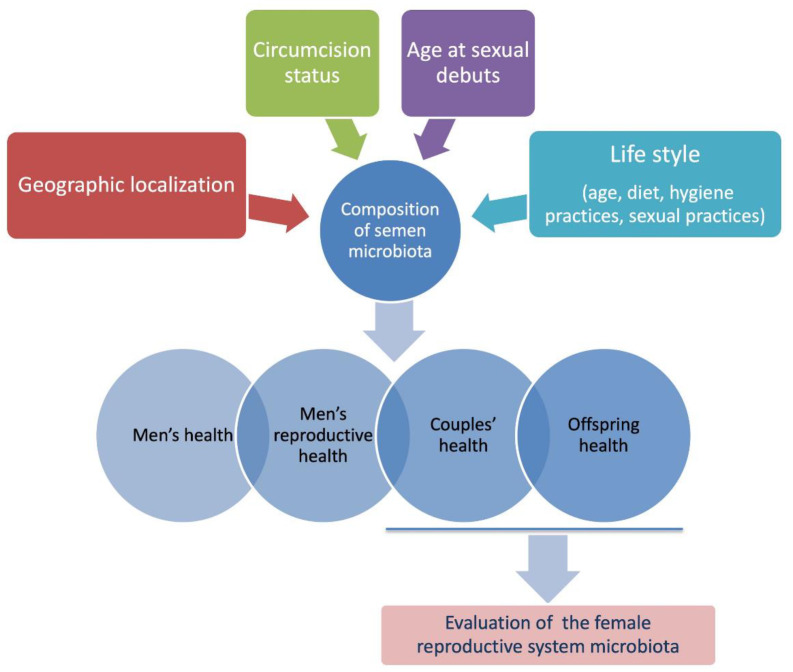
Individual variability factors affecting semen microbiota composition and processes in which semen microbiota has been suggested to play a role [12,19,21,23,36,49,55,56,57,58,63]. The simultaneous evaluation of the female partner’s microbiota should be taken into account considering their mutual influences and the potential effects on couples’ and offspring health [64].

Owing to the availability of high-resolution molecular methods that are able to investigate microbial communities’ richness and biodiversity, metagenomic studies have been prompted and microbes have been described in almost all body tissues and niches [1,2,3]. With regard to the male reproductive system, the semen microbiota has been described in humans and seems to play a role in healthy status maintenance [8,9,12]. Thus, semen microbiome dysbiosis may lead to diseases. Interestingly, increasing evidence is accumulating, highlighting a role of the semen microbiome in infertility [9,26,27,37,49,57,58]. However, as for other metagenomic studies, it is important to underline that several analytical and biological variables (i.e., patients’ selection, the geographical location, diet, age, hygiene practices, circumcision status, and sexual activities of the study participants, sampling methods and handling, sequencing strategies, statistical analyses) may affect the reliability of results, leading to interpretation biases and impairing their reproducibility (Figure 2).

Thus, standardized procedures are required to improve the comparability of data across different studies. Further studies are needed to address this issue in order to improve our knowledge regarding the semen microbiome and explore its manipulation as a tool for ameliorating the infertility outcomes of couples. In this context, it is important to underline once again that the simultaneous evaluation of the female partner’s microbiota is mandatory to improve couple’s reproductive choices [63].
